# A New Story on the Multidimensionality of the MSPSS: Validity of the Internal Structure through Bifactor ESEM

**DOI:** 10.3390/ijerph19020935

**Published:** 2022-01-14

**Authors:** César Merino-Soto, Alicia Boluarte Carbajal, Filiberto Toledano-Toledano, Laura A. Nabors, Miguel Ángel Núñez-Benítez

**Affiliations:** 1 Instituto de Investigacion de Psicologia, Universidad de San Martin de Porres, Lima 34, Peru; sikayax@yahoo.com.ar; 2 Facultad de Ciencias de la Salud, Universidad Cesar Vallejo—Lima Norte, Lima 39, Peru; aliciabolucar@gmail.com; 3 Unidad de Investigacion en Medicina Basada en Evidencias, Hospital Infantil de Mexico Federico Gomez Instituto Nacional de Salud, Dr. Marquez 162, Col. Doctores, Cuauhtemoc, Mexico City 06720, Mexico; 4 Unidad de Investigacion Sociomedica, Instituto Nacional de Rehabilitacion Luis Guillermo Ibarra Ibarra, Calzada Mexico-Xochimilco 289, Arenal de Guadalupe, Tlalpan, Mexico City 14389, Mexico; 5 School of Human Services, College of Education, Criminal Justice and Human Services, University of Cincinnati, Cincinnati, OH 45221-0068, USA; naborsla@ucmail.uc.edu; 6 Unidad de Medicina Familiar 31, Ermita Iztapalapa 1771, 8va Amp San Miguel, Iztapalapa, Mexico City 09837, Mexico; aquetzalli03@yahoo.com.mx

**Keywords:** social support, structural equation modeling, factor analysis, adolescents

## Abstract

The internal structure of the Multidimensional Scale of Perceived Social Support (MSPSS) in adolescents has been evaluated with some factorial analysis methodologies but not with bifactor exploratory structural equation modeling (ESEM), and possibly the inconsistency in the internal structure was dependent on these approaches. The objective of the study was to update evidence regarding its internal structure of MSPSS, by means of a detailed examination of its multidimensionality The participants were 460 adolescents from an educational institution in the Callao region, Lima, Peru. The structure was modeled using unidimensional, three-factor and bifactor models with confirmatory factor analysis (CFA) and ESEM approaches. The models showed good levels of fit, with the exception of the unidimensional model; however, the multidimensionality indicators supported the superiority of the bifactor ESEM. In contrast, the general factor was not strong enough, and the interfactorial correlations were substantially lower. It is concluded that the MSPSS can be interpreted by independent but moderately correlated factors, and there is possible systematic variance that potentially prevented the identification of a general factor.

## 1. Introduction

Adolescence is an intermediate stage between childhood and adulthood. Adolescence begins at the age of puberty, but it does not have an end age; it is fundamentally defined by psychological characteristics, and its duration is determined by the socioeconomic and cultural context in which the person lives [1,2]. Adolescence represents 18% of the total population in Latin America and the Caribbean [3] and 16% worldwide [4]. It is not only an evolutionary stage of great challenges but also a stage of high vulnerability and psychosocial risk [5], which can be evidenced by the presence of risky behaviors such as alcohol consumption in young people aged 15–19 years (27%), with an onset age of less than 15 years [6]. Likewise, data on drug use show a higher incidence in young people aged 18 to 24 than in adults [7]. The adolescent pregnancy rate worldwide is 46 births per 1000 girls [8], suicide was the second leading cause of death in individuals aged between 15 and 29 years in 2016 [9] and depression continues to be a problem in this population [10].

Adolescent development is determined by individual and socioenvironmental factors, and, from the point of view of the ecological model [11], it is not only family and friends who exert a shaping influence [12] but also contexts such as school and community. The construct of social support in adolescence has been studied from different perspectives and approaches and is predominantly considered a protective factor [5] that acts to prevent psychological disorders, such as depression, and their serious repercussions on adolescent health [13]. Social support has been studied as a predictive factor of social skills [14] and well-being [15] and a mediating variable of emotional intelligence [16,17], and it contributes to reaffirming feelings of belonging and personal worth in adolescents [18].

Although there is no unique definition of social support, there is a certain level of agreement that it involves interactions between two or more people through the provision of affection, care, material help, services, advice, and other useful information for the well-being of the person [18,19,20]. Classically, social support has been divided into two dimensions: one is related to the number of support networks that are established, and the other is the subjective characteristics of support perception, which can be affective, instrumental, and informative [21]. Recently, it has become necessary to deepen the analysis of social support to understand the problems of adolescents immersed in juvenile delinquency, drug use culture, school failure, and suicide, especially in this stage where peer groups often determine the positive or negative behavior of the adolescent [22].

Social support, a multidimensional and controversial construct at the same time, has been measured with a variety of instruments, such as the social support survey (MOS, 21 items; [23]), and applied in populations with different health conditions and in adolescents. The Family and Friends Social Support Scale (AFA-R, 14 items; [24]) is specifically aimed at adolescents and young people. Additionally, the Children and Adolescent Social Support Scale (CASSS, 40 items; [25]) assesses perceived social support from four sources: parents, teachers, classmates, and friends. Furthermore, the Social Support Networks Scale (SSNS) was developed for family caregivers of children with cancer [26]. Finally, the Multidimensional Scale of Perceived Social Support (MSPSS; [27]) was the target measure for the present study.

The MSPSS has demonstrated versatility because it has been applied in different contexts and age groups. It provides information regarding the subjective evaluation of social support obtained from sources that have been grouped into three main subscales: family, friends, and significant others [27]. Methodological studies carried out with the scale in the adolescent population have confirmed this three-dimensional factorial structure; however, the literature shows differences in the significant others’ dimension [21,28,29,30], and this dimension has shown a high correlation with the friends’ dimension, which makes it difficult for adolescents to discriminate between the two dimensions. The MSPSS has also shown a relationship with other study variables; for example, Edwards [31] found a correlation (r = 0.53, *p* < 0.05) between the family dimension with family support from the Familism Scale [32] and between the satisfaction dimension with the Multidimensional Students Life Satisfaction Scale (MSLSS; [33]; r = 0.79, *p* < 0.05). Similarly, Canty-Mitchell [34] compared the Adolescent Family Caring Scale (AFCS; r = 0.76, *p* < 0.05) to the other dimensions of the MSPSS. Another study reported that MSPSS scores had a positive correlation with resilience and negative correlations with depression, levels of exposure to violence, and childhood trauma [35]. Additionally, Navarro-Loli et al. [36] demonstrated through a linear regression analysis the predictive power of the MSPSS for depressive symptoms in adolescents. Ramaswamy et al. [37] demonstrated concurrent validity, finding a positive correlation with the assistance-seeking dimension of the Coping Scale for Children and Youth (CSCY; [38]) and a negative correlation with the internalizing behavior scale of the Youth Self-Report (YSR; [39]) and the Adolescent Daily Hassles Scale (ADHS; [40]) and concluding that more social support means fewer complaints from the school. This literature essentially points out the theoretical and convergent coherence of MSPSS scores with other variables. However, the literature on the internal structure of the MSPSS and how the internal structure influences the use of MSPSS scores appears to be less consistent.

For the purpose of identifying the pattern of results and methodological procedures used in the study of the internal structure of the MSPSS, a rapid systematic review was carried out [41,42] using systematic review methods. The following keywords were used, and only psychometric studies conducted with adolescents were included: MSPSS, social support, adolescence, validity, and reliability. The review was performed through search engines in the ScienceDirect, Scopus, PubMed, and Google Scholar databases. Because the objective of this study was focused on adolescents and the internal structure of MSPSS, the exclusion criteria were a youth-adult sample and nonpsychometric studies (e.g., [43]). Table 1 shows the results of the search.

Based on Table 1, the predominant model that was retained included three correlated factors; with the exception of the study by Trejos-Herrera et al. [46] that conducted a priori testing of this model. However, in an analytical context, confirmatory factor analysis (CFA) provides the advantage of directly testing and contrasting models; for example, it is possible to conduct a priori testing of a unidimensional, multidimensional and/or multidimensional bifactor model in the same study. These models are usually considered in the evaluation of the multidimensionality of a measure [48,49,50]. MSPSS total and subscale scores are typically reported, and correlations between these scores vary between moderate to high levels; these correlations are generally obtained using the maximum likelihood (ML) estimator, which is an analytical procedure that is not robust with nonnormal data [51,52].

On the other hand, the predominant factorial design was of a unique group, in which the internal structure parameters were estimated in a single sample without adding internal replicability options. The replicability of results in quantitative methodologies is important, however, only some studies implemented measurement invariance or sample partitioning. The sequential application of exploratory factor analysis (EFA) or CFA in the same sample cannot be considered a replicability test because what is obtained is a change in the estimation method or fit estimators that the EFA does not have. Some inconsistencies in these reports were also found, such as omitting the interfactoria correlations or identifying their source (observed or factorial scores), the estimation method, and the type of correlational matrix used (although not reported in Table 1, these can be polychoric or Pearson correlations). Regarding the estimation method, the principal component analysis (PCA) and varimax rotation have not yet disappeared in the psychometric methodology applied to the MSPSS. Finally, the internal consistency or reliability has been predominantly estimated by α coefficients, and the derived information may be underestimated due to limitations that were rarely recognized in the studies reviewed (the literature on this is extensive; for an example see [53]). Two additional problems that stand out were the coincidence with one of the observations of the MSPSS systematic review by Dambi et al. [43], that it was rare that a study contrasted several measurement models (e.g., one-, two-, or three-factor models), and that the relationship with social desirability was not explored (see Table 1).

An additional implication of this literature review is that the MSPSS is an instrument with numerous studies for evidence of internal structure and other sources of validity, and differences in internal structure were found. These differences in internal structure may be due to the method of assessing dimensionality, sample idiosyncrasies, cross-cultural differences, or biases not explicitly assessed (careless response). But a major source of these differences, keeping things equal, is the dimensionality assessment methodology. With the advancement in this methodology over the years, and in the context that to date the MSPSS dimensionality research has not been updated, the present study focuses on this point, that is, to introduce ESEM modeling into MSPSS dimensionality research.

Table 1 also shows that the MSPSS has not yet been assessed with other structure analysis models, for example, exploratory structural equation modeling (ESEM; [54]) and bifactor ESEM [55]. Both are advanced models that were introduced to overcome the problems of CFA modeling and to better evaluate the structural properties of multidimensional measurements. ESEM contains exploratory and confirmatory approaches, and the characteristic procedure consists of estimating the crossed or divergent factor loads with the other factors analyzed, not only the convergent factor (which is hypothesized as the causal influence of the items). This estimation has been shown to influence the decrease in factor loadings and interfactor correlations [54,56]. In this way, the factorial solutions obtained by the ESEM approach are considered realistic [54] because they estimate the parameters that by design are not estimated in the CFA (factorial loads in the non-hypothesized factors). On the other hand, the bifactor has been rediscovered as an effective method to test the multidimensionality of a measure [48,49,50,57,58]. It basically consists of modeling a general factor common to all items, together with specific factors that represent the unique content of the items [54]. Integrating both approaches into a single model (i.e., bifactor ESEM) has not yet been used in the structural analysis of the MSPSS, although this approach could provide new information on the internal structure and modify the interpretation of the scores previously evaluated by CFA models.

Therefore, an attempt is made to advance the research on the internal structure by implementing the ESEM approach. This analytical alternative could yield greater precision in realistic conditions of factorial complexity in multidimensional instruments [55]. Because the MSPSS tends to show moderate or high correlations between its dimensions (see Table 1), the bifactor ESEM model can distinguish the sources of systematic variance by estimating the relationship of the items with all the dimensions and not only with its hypothesized dimension; that is, this approach results in estimates of cross loads that are not usually estimated in CFA models [55], including a general factor that can represent the systematic common variance in the MSPSS. Thus, this study aligns with the importance of examining instruments in new contexts and analysis conditions, as provided by guidelines from influential documents that guide the practice of test adaptation [59].

## 2. Materials and Methods

### 2.1. Participants

The study sample came from the Callao region, one of the areas in Lima (Perú) with high rates of violence, citizen insecurity, drug use, pollution, and poverty [60]. Due to availability and internal organizational ease, a public educational institution was chosen. This institution enrolls males and females at the high-school level, between 12 and 16 years old [61] and mostly from families of low socioeconomic status. Its administrative organization is the same as other Peruvian public institutions. The total population was 1042 enrolled students. The units sampled were classrooms that were randomly selected by simple sampling. Each classroom contained approximately 20 to 35 students, with 15 classrooms being selected. The total sample drawn from this selection was 510 adolescents between the ages of 11 and 18 years. Several exclusion criteria were taken into account: students identified with a disability condition, as defined by the “Service of Support and Advice for the Attention of Special Educational Needs” (SAANE; RM No 665, 2018), foreigners, and those who did not agree to participate voluntarily. After applying the exclusion criteria on irrelevant response patterns and multivariate extreme values (see below), the effective sample was 461 participants.

### 2.2. Instrument

The MSPSS [27] measures the perceived support that the person can receive from family, friends, and significant others. It is composed of 12 items, grouped into three dimensions: family (FAM; items: 3, 4, 8 and 11), friends (FRI; items: 6, 7, 9 and 12), and significant others (SO; items: 1, 2, 5 and 10). Response options are on an ordinal scale (1 = most of the time, 2 = sometimes, 3 = often, 4 = always or almost always). The approximate duration to complete the questionnaire is 10 min. All the items have a positive orientation, and higher scores indicate greater social support. This study used the Spanish adaptation made by Arechavala and Miranda [62], who varied the response scaling from the original version to facilitate the adolescents’ level of understanding and avoid biases in the answer trends. This version was compared with the content of version validated in a previous Peruvian study. [36], which was based on the version provided by the author of the instrument; for consistency check, it was also compared with other recent Latin American versions [46].

### 2.3. Ethical Considerations

This study is a part of the research project (HIM/2015/017/SSA.1207; “Effects of mindfulness training on psychological distress and quality of life of the family caregiver”) that was approved on 16 December 2014, by the Research, Ethics, and Biosafety Commissions of the Hospital Infantil de México Federico Gómez, National Institute of Health, in Mexico City. While conducting this study, the ethical rules and considerations for research with humans currently enforced in Mexico [63] and those outlined by the American Psychological Association [64] were followed. All family caregivers were informed of the objectives and scope of the research and their rights in accordance with the Declaration of Helsinki [65]. The caregivers who agreed to participate in the study signed an informed consent letter. Participation in this study was voluntary and did not involve payment. The caregivers who provided consent for their child to participate completed an informed consent letter. Youths provided assent and returned a survey if they wished to participate.

### 2.4. Procedure

#### 2.4.1. Data Collection

Coordination was carried out with the principal’s office of the educational institution to obtain authorization to access the study sample, and the data were collected between June and August 2019. The informed consent form explaining the objective of the study had been previously delivered to parents through the tutors in the classrooms and requested the voluntary participation of their child. Adolescents whose parents agreed to participate filled out the informed consent form. No identifiable information on the participants was recorded. The scale was administered during the tutoring hour, and at the end of data collection, they were offered a talk on family functionality; completion of the process lasted approximately seven weeks. The entire procedure is aligned with the Declaration of Helsinki regarding anonymity, protection of responses and freedom of participation in the study.

#### 2.4.2. Data Analysis

First, before examining the internal structure, outliers and irrelevant response patterns were identified. As a general measure of abnormal responses [66], the D2 distance [67] was used for each subject based on the quadratic distance of the multivariate centroid of the variables (i.e., the MSPSS items). Based on the metrics of the *χ*^2^ distribution (degrees of freedom equal to the number of items), the Bonferroni correction [68] was applied at a 0.05 confidence level to establish the cutoff point for detection (*χ*^2^ > 6.14). The detection of response patterns associated with insufficient effort was done by estimating the sequence of the longest string of identical responses given by a person (*longstr*), which, in this context of multidimensional measurement, suggests responses without variability [66]. For this purpose, the careless R program was used [69]. Second, a descriptive analysis of the items was performed, which included their distributional characteristics (i.e., distributional normality) and differences in response trends. For each subscale, these were examined using nonparametric analyses that included a measure of the magnitude of the difference [70] using the langtest [71] and MVN [72] R programs.

**Figure 1:**
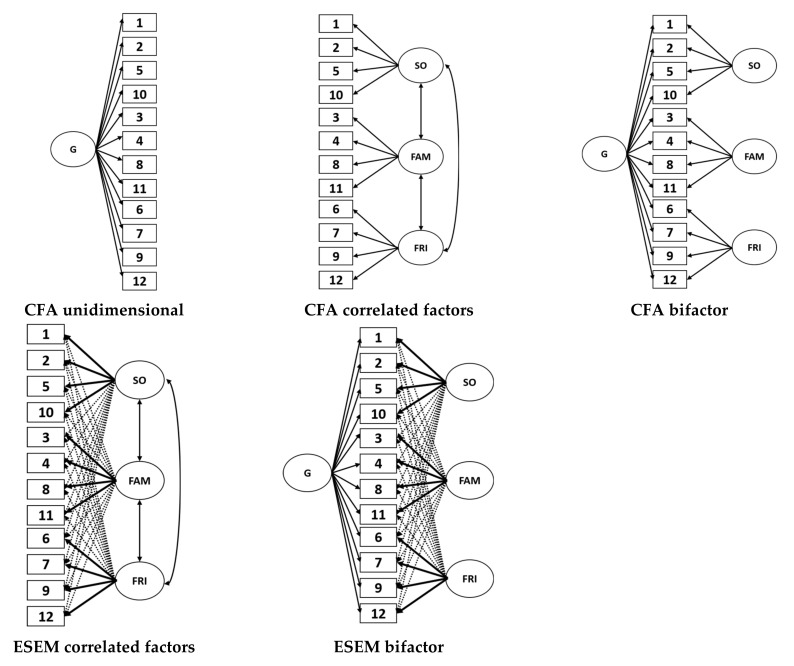
Representation of the CFA and ESEM models tested. G: General factor; SO: Others support factor; FAM: Family support factor; FRI: Friends’ support factor.

Third, the internal structure was evaluated through CFA-SEM to evaluate the MSPSS measurement model. First, the model established by the author Zimet et al. [27] and most subsequent studies, which consisted of three related dimensions (3F), were tested. The second model represented the use of the total MSPSS score, that is, it was defined by a single latent dimension (i.e., unidimensionality). The third model included bifactor modeling, which allows the identification of the general latent dimension (i.e., a general factor) and specific dimensions (i.e., specific factors). Because the literature generally finds moderate or strong associations between the dimensions of the MSPSS, ESEM modeling was applied in the model with three related factors (model 3F) and in the bifactor model. Both approaches (CFA and ESEM) can be applied in a complementary way to assess the multidimensionality of instruments. Oblique geomin rotation [56] was applied in the 3F-ESEM and bifactor-ESEM models. Due to its efficacy [51,52], the WLSMV estimator was used in all SEM modeling [73] with interitem polychoric correlations. Figure 1 shows the representation of each model tested.

In general, the fit of each of the models was evaluated using metrics such as CFI (≥0.95), RMSEA (≤0.05), WRMR (≤0.90; [74]) and gammaHat (≥0.95). For bifactor modeling (bifactor CFA and bifactor ESEM), several indicators suggested in the literature were used [49,50,58,75,76]: (a) the explained common variance (ECV) retained in the general factor Fg (ECVg), in the items of the Fg (I-ECV) and in the specific factors after removing the general variance (ECVf) had to exceed 0.70 or 0.80; (b) the reliable variance in the observed score (*ω_h_*) and in the specific factors after removing the variance in Fg from the observed score (*ωhs*) had a cutoff point of ≥0.80; and (c) the degree of replicability of the construct in each factor (H ≥ 0.80) and the absolute relative parameter bias (ARPB), which are in the 10% to 15% range for the difference between the factorial loads estimated in the general factor of a unidimensional model vs. a bifactor model, are acceptable [49,50]. Finally, the determinability of the factors (FD) was also estimated (FD > 0.90; [77]).

Furthermore, due to the predominance of the ECV in establishing the general bifactor dimension [78], ECV values higher than 0.70 suggest that there is an argument based on the common variance for the conclusion of an important unidimensional model [49,50]. An ECV below 0.70 suggests that the multidimensionality coming from the specific factors is nontrivial. The potential misspecifications were evaluated for each model using an approach that combines the statistical power and the size of the modification [79]. SEM modeling was carried out by the lavaan [80] and semtools [81] R programs.

Finally, based on Table 1, the interfactorial correlations were meta-analyzed using the random effects model, an appropriate method when the effect of the sampling error from different populations is presumed [82]. The Hartung–Knapp–Sidik–Jonkman estimator was used, since it can produce robust results with a small number of studies that have a high degree of heterogeneity [82,83].

## 3. Results

### 3.1. Response Bias Analysis

The relationship between the identification of D^2^ and *longsting* was −0.046 (*p* > 0.10), indicating that both measures detected different response patterns. Fourteen subjects (2.7%) with D^2^ > 41.78 exceeded the cutoff point (D^2^ ≥ 41.004) and were detected as multivariate outliers. Inspection of these responses revealed an inconsistent pattern of response. On the other hand, 35 subjects (6.8%) were identified with the same answers in all 12 items of the MSPSS, in categories 1 (1, 2.8%), 2 (1, 2.8%) and 3 (33, 95.2%). Altogether, 49 sample subjects (9.5%) were removed.

### 3.2. Descriptive and Correlational Analysis

#### 3.2.1. Item Analysis

The responses to the items (see Table 2) showed apparent similarities in the direction of their distributional properties (skewness and kurtosis with the same sign) but also showed apparent differences in magnitude. The global difference in the responses on the SO scale was trivial (Friedman-*χ*^2^ = 2.3247, gl = 3, *p* = 0.507; *r* = 0.031, 95% CI = −0.061, 0.122), while the differences on the FAM scale (Friedman-*χ*^2^ = 265.53, gl = 3, *p* < 0.001) and FRI scale (Friedman-*χ*^2^ = 38.83, gl = 3, *p* < 0.001) were statistically significant but small in size (*r* = 0.191, 95% CI: 0.101, 0.277; and *r* = 0.249, 95% CI = 0.166, 0.329, respectively). Furthermore, the responses to the items were not distributed with statistical normality (see Cramer–von Mises test statistics for each item), and multivariate normality, based on the Henze and Zirkler [84] test, was not fulfilled in the set of items, *z* = 1.77942 (*p* < 0.001).

#### 3.2.2. Meta-Analytic Interfactor Correlations

Using the CFA studies in Table 1 (studies 3, 4, 5, 8, 9 and 10), the meta-analytic interfactorial correlation between FRI and SO was 0.56 (*p* < 0.01, 95% CI = 0.38, 0.73), the interfactorial correlation between FRI and FAM was 0.39 (*p* < 0.01, 95% CI = 0.29, 0.48), and the interfactorial correlation between SO and FAM was 0.51 (*p* < 0.01, 95% CI = 0.54, 0.70). The estimates of variability for the FRI and SO relationship (*Q* = 625.69, *p* < 0.01, *τ* = 0.215, *I*^2^ = 98.92%, *H*^2^ = 95.53), FRI and FAM relationship (*Q* = 85.39, *p* <.01, *τ* = 0.115, *I^2^* = 93.42%, *H*^2^ = 15.20), and FAM and SO relationship (*Q* = 204.713, *p* < 0.01, *τ* = 0.146, *I^2^* = 96.91%, *H*^2^ = 32.37) indicated heterogeneity in the correlations that integrated the meta-analytic average. These meta-analytic interfactor correlations were used for comparison with the interfactor correlations in our sample (see Section 3.3.3).

### 3.3. Internal Structure

#### 3.3.1. Model Fits

The general results in Table 3 indicated that the unidimensional model was of comparatively lesser statistical fit. Additionally, with both approaches (CFA and ESEM), the correlated three-factor model (congeneric model) fitted very well, but the bifactor model quantitatively outperformed it. The difference between the two approaches does not seem statistically substantial because both expressed a very good fit. The performance of the ESEM model was definitely superior to that of the CFA model, indicating that the estimation of the cross loads, as usually occurs in the ESEM, did have an impact on the amount of fit obtained.

#### 3.3.2. CFA Modeling

Inspection of the parameters of interest in the CFA models (Table 4), with the exception of the bifactor-CFA model, revealed that the factor loadings were high in all models (λ > 0.70). In contrast, in the unidimensional model, although its fit was poor (see previous paragraph), its factor loadings were high (>0.66). In the 3-factor model, the interfactorial correlations were high (>0.60), suggesting insufficient discrimination between them. The bifactor-CFA model did not initially converge, and adjustment indicators could not be obtained. The apparent problem was item two of the SO factor, which showed excessive negative variance (=−678.20), and this factor load was identified as a Heywood case in its specific factor (λ = 26.04). Its load in the general factor (F_g-cfa_) and the other items of its factor showed loads λ > 0.80. Because the SO factor presented similar factorial loads (tau-equivalence; see next paragraph), its loads were constrained to equality between them to control the negative variance.

As a complementary analysis for each factor, their factor loadings were constrained to equality (tau-equivalent model), while the remaining factors were freely estimated (congeneric models). Compared with the model that had free estimation of all factorial loads (three correlated factors), the results showed a good fit for the SO (WLSMV-*χ*^2^ = 70.802, *p* > 0.05, gl = 54, CFI = 1.00, Δ_CFI_ = 0.000; WRMR = 0.788, Δ_WRMR_ = −0.019; gammaHat = 0.993, Δ_gamma_ = 0.001) and FRI factors (WLSMV-*χ*^2^ = 83.593, *p* < 0.05, gl = 54, CFI = 0.999, Δ_CFI_ = 0.001; WRMR = 0.856, Δ_WRMR_ = −0.087; gammaHat = 0.989, Δ_gamma_ = 0.005). The tau-equivalent fit was acceptable for the FAM factor (WLSMV-*χ*^2^ = 199.502, *p* < 0.01, gl = 54, CFI = 0.997, Δ_CFI_ = 0.003; WRMR = 1.323, Δ_WRMR_ = −0.554; *gammaHat* = 0.950; Δ_gamma_ = 0.044). The estimated values of the fixed factorial load in the SO factor (λ = 0.843), FAM factor (λ = 0.853) and FRI factor (λ = 0.901) were very high.

In the analysis of the multidimensionality of the MSPSS with the bifactor CFA, the ECV indicators, *ω_h_* and H, showed the following: (a) the ECVg indicator of the general factor Fg retained a moderately high variance (ECV_g_ = 0.751) and was highly differentiated compared with the specific factors (ECV_f_ around 0.35), and at the item level (I-ECV), the general common variance was moderate in the FAM and FRI factor items, and high for the SO factor items; (b) the items were shown to be predominantly within the acceptable range of multidimensional bias (between 0.10 and 0.15; [50]); (c) the reliable variance (*ω_h_*) was high for Fg (>0.80) and low for the specific factors (<0.40); (d) the coefficient (H) indicated that the replicability was stronger for the general factor (H > 0.90) than for the specific factors (H < 0.70); (e) the determinability of the general factor Fg exceeded the minimum criterion (FD > 0.90) compared with the specific factors (FD < 0.90); and (f) ARPB occurred in a range that suggested that the general dimension Fg does not produce significant biases regarding the multidimensionality of the items (ARPB _total_ = 0.121). Taken together, the CFA modeling showed that the bifactor model represents a dimensional solution where a general factor Fg is predominant and preferable over the consideration of specific factors as dimensions to interpret. The interfactor correlations could not be estimated due to the orthogonality constraint to run the CFA bifactor.

#### 3.3.3. ESEM Modeling

Table 5 shows the results of the ESEM modeling. In the 3F-ESEM model, items with factorial complexity were found (divergent or crossed loads, λ ≥ 0.10; items 3, 6, 8, 10, 11), and these were contrasted with the CFA specification on this type of factor loading (hypothesized factor loads equal to zero). Compared with the 3F-CFA model (Table 4), the interfactor correlations were comparatively low. Comparing the meta-analytic correlations with the correlations in 3F-ESEM, the differences were statistically significant and comparatively substantial in FAM-SO (*z* = 9.63, *p* = 0.01, *q* = 0.45), SO-FRI (*z* = 7.18, *p* = 0.01, *q* = 0.33) and FAM-FRI (*z* = 6.05, *p* = 0.01, *q* = 0.28).

The bifactor ESEM model produced very different interfactor correlations than the 3F-ESEM, because they decreased between 25.2% and 65.2%. Compared with the meta-analytic correlations, statistically significant but small differences were found in FAM-SO (*z* = 1.94, *p* = 0.02, *q* = 0.09), SO-FRI (*z* = −4.88, *p* < 0.01, *q* = 0.22) and FAM-FRI (*z* = −4.27, *p* < 0.01, *q* = 0.19). However, factor loadings decreased in different proportions, sometimes by a moderately high amount, within the same factor: SO between 2.4% and 16.1%, FAM between 2.0% and 8.3%, and FRI between 4.7% and 14.8%. These decreases were more accentuated with the implementation of the bifactor ESEM.

In the multidimensional analysis, the bifactor ESEM produced values of the ECV, *ω_h_*, H and ARPB with the following characteristics: (a) the ECV_g_ indicator of the general factor Fg was low compared to the recommended criterion, and that of the subscales was even lower (ECV_f_ between 0.10 and 0.24), while at the item level (I-ECV), the general common variance was high only for FRI and low for FAM and SO; (b) the items showed a predominant multidimensional bias (ARPB_item_ > 0.15) in SO and FAM; (c) the reliable variance (*ω_h_*) for Fg was acceptable, and low for the specific factors (<0.10); (d) the coefficient (H) indicated that replicability was stronger with the general factor (H > 0.90) than with the specific factors; (e) the determinability (FD) of the general factor Fg and FAM exceeded the minimum criterion (FD > 0.90), however, it was moderately low for the other factors (SO and FRI); and (f) the degree of bias (ARPB _total_ = 0.238) occurred in a range that suggested nontrivial differentiation between the factorial loads produced from a general dimension *Fg* and the multidimensional model. Altogether, ARPB (at the total and item level) and ECV for the general factor (ECV_total_) and the items (I-ECV) of the bifactor ESEM model showed that the general factor was not strong enough.

## 4. Discussion

In the evolution of the MSPSS, a study introduced CFA to evaluate its internal structure [44] and achieved an advance in the direct verification of its dimensional models (e.g., first-order and second-order factors) and of the equivalence of these. In this study, bifactor ESEM model was introduced for the first time to examine the internal structure of the MSPSS, and the results indicated a significant contrast with all previous studies that applied CFA.

Regarding factor loadings, they decreased across a mixed range of changes, indicating that the validity of each item was differentially affected by the model used. The greatest decrease occurred with the bifactor ESEM, which is aligned with the frequent results found with the ESEM modeling in general and with the bifactor ESEM in particular [55,56,57]. Another result to be emphasized is that CFA concealed some misspecifications associated with nontrivial cross-factorial loads. As is known in the methodological literature [55], nonrealistic restrictions are imposed with CFA, and this is the main reason why ESEM models tend to show a better statistical fit than CFA models.

The *ω_h_* obtained for the general factor (0.795) indicated that there is enough reliable variance in the total score to be interpreted, and H suggested that the greater replicability and better definition obtained in the estimation of a general factor compared with the specific factors was beyond doubt. On the other hand, H suggested that the definition of the factor from its items is poor for FRI and moderate for SO and FAM. Both the potential replicability and the high definition of the constructs are derived from the interpretation of H [75]. Finally, based on the ECV for the factors, the individual differences can be captured by the general factor and FAM, and moderately by SO. With an explained variance in ECV = 0.795, 79.5% represents the common variability source, and this amount cannot be considered trivial. Therefore, in conjunction with the determinability of the factorial solution, reliable variance, and replicability (FD coefficients, *ω_h_*, and H, respectively), it is tempting to accept the interpretation of a general factor even in the context of the ECV being below the recommended cutoff point (<0.70).

However, the complementary information at the item level (I-ECV and I-ARPB) suggested that the strength of the potential general factor did not guarantee accepting it as representative of a global construct of social support. Because the ECV is one of the key pieces to accept a bifactor model [78], its results at the item and general factor levels contrast with the rest of the general indices, it can be affirmed that there is a partial confirmation to accept the general factor; in addition, the parameters at the item level (i.e., I-ECV and I-ARPB) predominantly indicated a difference between multidimensionality and unidimensionality that cannot be ignored. One of the risks of accepting unidimensionality when I-ECV is less than 0.85 is the violation of local independence [85], and this is possibly more certain when half of the I-ECV values met the recommended cutoff point (>0.80).

The results of the bifactor CFA contrasted with the bifactor ESEM in the estimation of interfactorial correlations and in the conclusion about multidimensionality. In the bifactor CFA, the conclusion of unidimensionality seemed reasonable; however, this was shown to be overestimated when the structure of the MSPSS was evaluated by the bifactor ESEM. It was in this analysis that the general factor was not strong enough, and contradictory indications of multidimensionality were obtained. On the other hand, the interfactorial correlations reported by the bifactor ESEM were more differentiated among themselves compared with the rest of the factorial results, especially with the bifactor CFA. This differentiation adds an additional advantage in relation to the precision of these correlations because they realistically add the estimation of all factorial loadings of all the factors analyzed, which is characteristic of ESEM [54].

As observed, the interfactorial correlations estimated in the bifactor ESEM model can be interpreted as a structure with moderate or low conceptual dependence, and this has acceptability for the construction of instruments such as the MSPSS when measures that do not conceptually overlap are required. A large dependence between factors usually indicates insufficient conceptual discrimination between them [76,85], and to this same extent, the interpretation is factorially complex. Instead, the amount of dependence found in the bifactor ESEM may be more satisfactory and in line with what is found in other studies with different instruments (e.g., AFA-R: [24]; CASSS: [25]; MOS: [23]). In these studies, the support sources do not conclude that there is a strong degree of dependence between them, which would lead to potential problems of divergent validity in the instrument. Although it is plausible that social support sources covary in a subject, it is more reasonable to rely on the idea that they act independently unless the context of the person links them. Also, the multidimensionality of adolescent responses to MSPSS indicated that there is a need to examine perceptions of support in different areas, such as support of friends and significant others. Assessment of adolescents’ perceptions of different types of support may be critical to the design of prevention messaging and interventions to improve their functioning.

It is possible that the specific undetected variance from undetected processes is involved in the variance in the general factor, inflating the parameters of interest (e.g., factor loadings). If this is so, this general factor can represent the joint effect of careless responses, composed of average responses and social desirability [86,87], method variance or the interaction between these. As has been detected in other studies [66,86,87,88], this type of variance is omnipresent in measurements based on self-report and may be incorporated in the associated systematic variance in the content of the MSPSS.

The essential limitations of this study correspond to examining the invariance of the best fitted models (3F-ESEM and bifactor-ESEM models), as well as introducing methods that model the possible effect of irrelevant response styles. It is also important to point out sample size as an issue, because a larger size may be required to guarantee more stable results and a higher density in the groups compared for invariance measurements. However, our sample size is considered adequate, given the high trend in magnitude of the factor loadings (essentially > 0.60). Also, we did not have a measure assessing whether social desirability in the responding of the adolescents changed how items were characterized or reported. It may be beneficial to add a measure or questions to detect this in future studies. Although there is a report indicating that social desirability is not involved in MSPSS responses [89], for purposes of replicability and consistency in conclusions, this potential effect has yet to be studied. Finally, the chosen sample of participants does not ensure population representativeness, because this lack of representativeness may involve a range of experiences in social support that differ between low and other socioeconomic statuses.

The results introduced several implications for subsequent psychometric studies of the MSPSS, especially when examining its internal structure. One of the implications of the study is methodological. The distinction between the CFA and ESEM modeling was notable in the estimation of the parameters of interest (i.e., factor loadings and interfactor correlations) and consequently modified the conclusions about the conceptual relationships inferred from them. Both types of modeling provided two different figures to identify the best structural model that represented the structure of the MSPSS. In an analytical or methodological context, it is assumed that the implementation of both in a study can be considered canonical rather than complementary. Given the advance that ESEM modeling provides in understanding the internal structure of the MSPSS, it might be preferable to first choose the ESEM method to reveal the sources of variance in the items with better precision. Because it is common, reasonable, and realistic to find multidimensional variance in psychosocial measurement items [86], the ESEM effectively models this variance without imposing the known restrictions of the CFA.

Another implication is the requirement to consider modeling sources of systematic variance associated with irrelevant patterns or response styles that are particularly present in self-report measures (e.g., [66,87]), which are considered responses that are not necessarily related to the substantive content of the items. Some of these were detected in the study (i.e., extreme multivariate responses and long response sequences), representing a prevalence within the range reported in the literature (between 3.5% and 12%; [66]). However, an approach based on modeling can detect the variability in this phenomenon and look for other patterns, such as average responses and social desirability [88,89].

## 5. Conclusions

The present study highlights the effects of evaluation methods of the internal structure of the MSPSS carried out in previous studies and presents a more appropriate analysis of the multidimensionality or unidimensionality of the MSPSS in Peruvian adolescents. The MSPSS can be treated as a multidimensional measure, with its moderately associated dimensions in adolescents. However, the existence of a general dimension that is interpretable and expressed as a general score is not sufficiently justified because the general dimension was not psychometrically strong. These results, however, varied significantly as a consequence of the method of analysis of the internal structure used: with CFA modeling, a general dimension was recognized, but with ESEM modeling, the overall dimension was not strong enough. Additionally, with the CFA, there was a tendency to overestimate factorial loads and interfactor correlations. These overestimated interfactor correlations indicated a potential overall factor, with high strength to be treated as the single and representative MSPSS score. In contrast, ESEM modeling discovered moderate associations between the subscales and thus weakness of an overall factor. This finding is important, and points to the value of understanding adolescent perceptions of different types of support from family, significant others, and friends. Understanding the value and direction of supports needed from these different sources can be of value in designing interventions to improve social support of youth. Due to the limitations of the sample size in some interest groups, the equivalence of these results in the groups based on sex, age, and other groups requires investigation, as does a determination of the relationship of the MSPSS scores with external variables.

## Figures and Tables

**Table 1:** Psychometric studies in adolescent samples.

Version and Country	Participants	Factor Analysis Design	Factorial Configuration	Method	Factor Relationship	Internal Consistency	Invariance
1. Chou [28] Hong Kong	*N* = 475 Age: 17–18	1° PCA	2 factors: FAM: 9, 10, 11, 12 FRI: 1, 2, 3, 4, 5, 6, 7, 8	Estimator: NR Rotation: Varimax	FRI and others = NR FRI and FAM: 0.23 FAM and SO: NR	α: FRI: 0.94 FAM: 0.86 SO: NR	NR
2. Canty-Mitchell et al. [34] EEUU	*N* = 196 Age: M = 15.8 SD = 0.97	1° EFA	3 factors: FAM: 3, 4, 8, 11 FRI: 6, 7, 9, 12 SO: 1, 2, 5, 10	Estimator: Principal axis Rotation: Oblique	FRI and SO = 0.66 FRI and FAM: 0.53 FAM and SO: NR	α: FRI: 0.89 FAM: 0.91 SO: 0.91	NR
3. Cheng and Chan [44] Hong Kong	*N* = 2105 Age: M = 14.8 SD = 1.58	1° CFA	3 factors: FAM: 3, 4, 8, 11 FRI: 6, 7, 9, 12 SO: 1, 2, 5, 10 2 factors: F1: 6, 7, 9, 12, 1, 2, 5, 10 F2: 3, 4, 8, 11	Estimator: ML Model: 3 and 2 factors	FRI and SO = 0.85 FRI and FAM: 0.35 FAM and SO: 0.42	α: FRI: 0.76 FAM: 0.78 SO: 0.69	Age and sex Configuration
4. Bruwer et al. [35] South Africa	*N* = 502 Age: 11–23	1° CFA	3 factors: FAM: 3, 4, 8, 11 FRI: 6, 7, 9, 12 SO: 1, 2, 5, 10	Estimator: WLSMV Model: 3 factors	FRI and SO = 0.676 FRI and FAM: 0.616 FAM and SO: 0.747	α: FRI: 0.86 FAM: 0.86 SO: 0.88	NR
5. Ramaswamy et al. [37] EEUU	*N* = 635 Age: 11–15	1° CFA	3 factors: FAM: 3, 4, 8, 11 FRI: 6, 7, 9, 12 SO: 1, 2, 5, 10	Estimator: ML Model: 3 factors	FRI and SO = 0.30 FRI and FAM: 0.27 FAM and SO: 0.35	α: FRI: 0.75 FAM: 0.63 SO: 0.72	NR
6. Edwards [31] EEUU	*N* = 290 Age: 11–18	1° PCA	3 factors: FAM: 3, 4, 8, 11 FRI: 6, 7, 9, 12 SO: 1, 2, 5, 10	Estimator: NR Rotation: Varimax	NR	α: FAM: 0.88 FRI: 0.90 SO: 0.86	NR
7. Mosqueda et al. [22] Chile	*N* = 247 Age: 14–19	1° PCA	3 factors: FAM: 3, 4, 8, 11 FRI: 6, 7, 9, 12 SO: 1, 2, 5, 10	Estimator: NR Rotation: Varimax	NR	α: FAM: 0.850 FRI: 0.887 SO: 0.786	NR
8. Wilson et al. [45] Ghana	*N* = 717 Age: 15–18	1° CFA; 2° EFA	3 factors: FAM: 3, 4, 8, 11 FRI: 6, 7, 9, 12, 10 SO: 1, 2, 5	CFA Estimator: MLR Model: 3 factors EFA Estimator: ML Rotation: NR	FRI and SO: 0.35 FRI and FAM: 0.37 FAM and SO: 0.50	α: FAM: 0.73 FRI: 0.61 SO: 0.74	Sex: Metric
9. Trejos-Herrera et al. [46] Colombia	*N* = 763 Age: 14–18	1° CFA; 2° EFA	3 factors: FAM: 3, 4, 8, 11 FRI: 6, 7, 9, 12 SO: 1, 2, 5, 10	CFA Estimator: ML Model: 7 models EFA Estimator: ML Rotation: NR	FRI and SO: 0.46 FRI and FAM: 0.33 FAM and SO: 0.42	α: FAM: 0.82 FRI: 0.84 SO: 0.75	NR
10. Navarro-Loli et al. [30] Perú	*N* = 242 Age: 12–16	CFA	3 factors: FAM: 3, 4, 8, 11 FRI: 6, 7, 9, 12 SO: 1, 2, 5, 10	Estimator: Robust ML Model: 3 factors Bifactor	FRI and SO = 0.69 FRI and FAM: 0.37 FAM and SO: 0.62	α: FAM: 0.814 FRI: 0.874 SO: 0.824 Omega: (>0.85) FAM: NR FRI: NR SO: NR	NR
11. Aloba et al. [47] Nigeria	*N* = 1335 Age: 13–18	CFA	3 factors: FAM: 5, 6, 7, 8 FRI: 9, 10, 11, 12 SO: 1, 2, 3, 4 2 factors: FAM and SO: 1, 2, 3, 4, 5, 6, 7, 8 Am.: 9, 10, 11, 12	Estimator: ML Model: 3 factors and Hierarchical	FRI and SO = 0.65 FRI and FAM. = 0.67 FAM and SO = 0.82	α: FAM = 0.82 SO = 0.80 FRI = 0.78	Sex scalar
12. Okki et al. [29] Indonesia	*N* = 299 Age: 12–18	CFA	3 factors: FAM.: 3, 4, 8, 11 FRI.: 6, 7, 9, 12 SO: 1, 2, 5	Estimator: ML Model: 3 factors	FRI. and SO = 0.79 FRI. and FAM = 0.62 FAM. and SO = 0.71	α: FAM. = 0.81 FRI. = 0.78 SO = 0.79	Sex: scalar

Note. NR = does not report. EFA = exploratory factorial analysis. CFA = confirmatory factorial analysis. PCA: principal components analysis. FAM: Family support dimension. SO: significative others dimension. FRI: Friend support dimension.

**Table 2:** Descriptive statistics for the MSPSS items (*n* = 461).

	M	SD	Sk	Ku	CVM ^a^	Spearman’s Correlation ^b^	
						Sex	Age
mps1	3.461	1.150	−0.302	−0.823	2.754	0.058	−0.115 *
mps2	3.407	1.297	−0.341	−1.006	2.538	0.081	−0.068
mps5	3.431	1.259	−0.313	−0.984	2.550	0.007	−0.082
mps10	3.424	1.269	−0.320	−0.975	2.515	0.042	−0.058
mps3	3.827	1.052	−0.617	−0.339	3.675	−0.100 *	−0.174 **
mps4	3.662	1.167	−0.495	−0.772	3.256	−0.071	−0.193 **
mps8	3.063	1.256	−0.079	−1.023	2.239	−0.077	−0.142 **
mps11	3.355	1.282	−0.260	−1.053	2.460	−0.055	−0.141 **
mps6	3.363	1.173	−0.085	−1.000	2.634	0.129 **	−0.025
mps7	3.392	1.233	−0.162	−1.040	2.590	0.073	−0.018
mps9	3.342	1.353	−0.181	−1.222	2.671	0.110 *	−0.035
mps12	3.159	1.379	−0.096	−1.232	2.236	0.092 *	0.004

Note. CVM: Cramer–von Mises univariate normality test. ^a^ All are *p* < 0.001. ^b^ Correlations equal to or higher than 0.10: *p* < 0.05. Sk and Ku: skew and kurtosis coefficients. * *p* < 0.05. ** *p* < 0.01

**Table 3:** Fit indicators of the main tested models.

	CFA						ESEM			
	3 Factors		One Factor		Bifactor		3 Factors		Bifactor	
WLSMV-*χ*^2^	67.399		942.207		51.571		19.800		11.605	
df	51		54		45		60		56	
CFI	1.00		0.979		1.00		1.00		1.00	
WRMR	0.769		2.875		0.672		0.417		0.319	
RMSEA	0.026		0.189		0.018		0.00		0.00	
IC 90%	(0.00)	(0.042)	(0.179)	(0.200)	(0.00)	(0.03)	(0.00)	(0.00)	(0.00)	(0.00)
*GamaHat*	0.994		0.759		0.997		1.0		1.0	

Note. CFA: confirmatory factorial analysis. ESEM: exploratory structural equation modeling.

**Table 4:** Measurement models evaluated in the MSPSS using CFA (*n* = 461).

	**3 Factors**			**1 Factor**	**Bifactor—CFA ^a^**					
	**SO**	**FAM**	**FRI**		**Fg**	**SO**	**FAM**	**FRI**	**ICEV**	**ARPB**
mps1	0.838			0.780	0.826	0.145			0.970	0.056
mps2	0.861			0.804	0.848	0.136			0.975	0.052
mps5	0.836			0.780	0.823	0.146			0.969	0.052
mps10	0.834			0.775	0.821	0.146			0.969	0.056
mps3		0.890		0.807	0.728		0.463		0.712	0.109
mps4		0.928		0.847	0.727		0.627		0.573	0.165
mps8		0.747		0.674	0.582		0.483		0.592	0.158
mps11		0.784		0.710	0.627		0.461		0.649	0.132
mps6			0.880	0.832	0.740			0.453	0.727	0.124
mps7			0.894	0.853	0.736			0.506	0.679	0.159
mps9			0.935	0.894	0.765			0.530	0.676	0.169
mps12			0.888	0.854	0.698			0.583	0.589	0.223
Correlations										
SO	1				-	-	-			
FAM	0.785	1			-	-	-			
FRI	0.805	0.652	1		-	-	-			
Bifactor indicators										
ECV	-	-	-	-	0.751	0.029	0.370	0.334	-	-
FD	-	-	-	-	0.956	0.284	0.880	0.859	-	-
*ω*_h_	-	-	-	-	0.871	0.026	0.334	0.314	-	-
H	-	-	-	-	0.945	0.077	0.599	0.601	-	-

Note. SO: significant others. FAM: family. FRI: friends. GF: general factor. CEV: common explained variance. *ω*_h_: omega coefficient with the variance retained in the own analyzed factor. H: replicability coefficient. FD: factor determinability. ^a^ Bifactor-CFA model estimated with tau equivalence in the SO factor.

**Table 5:** Measurement models evaluated in the MSPSS using ESEM (*n* = 461).

	3 Factors			Bifactor—ESEM					
	SO	FAM	FRI	Fg	SO	FAM	FRI	I-ECV	I-ARPB
mps1	0.703	0.099	0.058	0.611	0.472	0.084	0.078	0.613	0.277
mps2	0.894	0.023	0.042	0.600	0.623	0.022	0.057	0.479	0.340
mps5	0.816	0.012	0.045	0.674	0.546	0.019	0.066	0.600	0.157
mps10	0.725	0.021	0.147	0.703	0.483	0.035	0.040	0.677	0.102
mps3	0.051	0.816	0.141	0.602	0.028	0.624	0.070	0.478	0.341
mps4	0.013	0.985	0.036	0.552	0.002	0.777	0.052	0.334	0.534
mps8	0.230	0.727	0.234	0.396	0.168	0.576	0.048	0.302	0.702
mps11	0.127	0.800	0.129	0.604	0.111	0.622	0.102	0.471	0.175
mps6	0.135	0.019	0.750	0.767	0.075	0.002	0.372	0.803	0.085
mps7	0.044	0.051	0.826	0.750	0.006	0.033	0.537	0.660	0.137
mps9	0.019	0.071	0.871	0.889	0.019	0.019	0.296	0.899	0.006
mps12	0.015	0.050	0.930	0.858	0.020	0.079	0.324	0.868	0.005
Correlations									
SO	1				1				
FAM	0.767	1			0.574	1			
FRI	0.748	0.601	1		0.384	0.209	1		
Bifactor indicators									
ECV	-	-	-	0.608	0.177	0.238	0.106		
FD	-	-	-	0.936	0.840	0.924	0.712		
*ω*_h_	-	-	-	0.795	0.059	0.085	0.029		
H	-	-	-	0.933	0.629	0.768	0.449		

Note. SO: significant others. FAM: family. FRI: friends. Fg: general factor. ECV: common explained variance. *ω*_h_: omega coefficient with the variance retained in the own analyzed factor. H: replicability coefficient. I-ECV: explained common variance in item level. I-ARPB: absolute relative parameter bias in item level. FD: factor determinability.

## Data Availability

The raw data supporting the conclusions of this article will be made available by the authors, without undue reservation.
